# Notes of Selected Cases from the Wards of the Charity Hospital, at New Orleans

**Published:** 1860-07

**Authors:** T. G. Richardson

**Affiliations:** One of the Attending Surgeons, and Professor of Anatomy in the Medical Department of the University of Louisiana


ART. I.—*Notes of Selected Cases from the Wards of the Charity Hospital, at New Orleans.* By T. G. RICHARDSON, M.D., one of the Attending Surgeons, and Professor of Anatomy in the Medical Department of the University of Louisiana.

*Dislocation of the Head of the Femur into the Thyroid Foramen, of eight days standing, reduced by Manipulation.*

W. S., aged twenty-six, a very stout, athletic man, was admitted into the hospital on the first of November, 1859, having been injured eight days previously by falling from the side of a schooner into a skiff. Attention being directed to the left hip, only a very slight examination sufficed to determine the nature of the accident. The patient lying upon his back, the leg was found to be flexed to a right angle at the knee-joint, the thigh strongly abducted and everted, and the limb lengthened about an inch and a half; in fine, all the signs of dislocation of the head of the femur into the thyroid foramen were well marked.

Determined to attempt reduction by manipulation, I had the man carried into the amphitheatre before the class and brought fully under the influence of chloroform. Complete insensibility and muscular relaxation having been effected, I seized the limb just above the ankle, flexed the leg still further at the knee-joint and the thigh upon the pelvis, bearing the latter down upon the left side of the abdomen, then carried the knee over to the middle line of the abdomen, and rapidly extended the limb, drawing it at the same time obliquely underneath its opposite fellow. Just before the extension, which required the exertion of a good deal of force, was completed, the limb suddenly yielded and fell into its natural position, but a single manipulation having been employed. The usual after-treatment was instituted, and in ten days time the patient, being able to walk with little or no difficulty, left the hospital.

Notwithstanding the readiness and comparative ease by which reduction was accomplished in this case, I am not prepared to recommend manipulation as a substitute in all cases for the old method of extension by the means of pulleys. It is without doubt a great improvement in the surgical art, but when abused, as it oftentimes is, it is liable to do great mischief to the soft parts around the joint. We read of practitioners who, in their efforts to accomplish reduction in this manner, have thrown the head of the bone from one position to another; say from the dorsum of the ilium into the sciatic notch, from the latter into the thyroid hole, and then back again upon the ilium. When this happens, great laceration and bruising of the soft parts must, of course, be produced, and hence the necessity for an accurate knowledge of the mechanism of the procedure and unusual care in its application. In reduction by the pulleys, the force being applied only in the direction of the displacement, no such accident can be produced. When the knowledge referred to is not possessed by the operator, I would advise adherence to the old method.

### *Concussion, with Laceration of the Brain and slight Extravasation of Blood; Death; Autopsy.*

An Irishman, name unknown, aged about thirty, was brought into the hospital on the afternoon of October 11, 1859, in a state of complete insensibility from an injury to the head received an hour previously. The history of the case, as gathered from those who witnessed the accident, was substantially as follows: The man was standing on the levee near the river's edge when a spar, extending from an adjacent boat to the shore, suddenly gave way, flew up, and struck him under the jaw with such force as to raise him entirely from the ground and throw him several feet distant. He was picked up in an apparently lifeless condition and conveyed immediately to the hospital, where I saw him the following morning.

Upon making inquiries of the student of the ward, I ascertained that the man was completely insensible when admitted, and that his vital powers were so much prostrated that the house surgeon deemed it advisable to administer brandy and water, which was gotten down only with great difficulty. Partial reaction followed the use of the stimulant, but up to the time of my visit, about twenty hours after the accident, there was no evidence of returning consciousness. Upon examination, I discovered the mark of the blow just below the lower jaw on the left side, but the skin was not lacerated nor the bone broken. His intellectual functions were completely suspended, nor could he be in the least aroused, or even made to open his eyes, by forcible shaking or by hollowing in his ears. His breathing was natural, pulse a little accelerated, and extremities warm. He had not vomited since his admission, but passed his urine involuntarily. The pupil of the left eye was slightly dilated, but the right was of its natural size, and responded readily to the influence of light.

While making this examination, I noticed that he was continually moving the left leg and arm, but that only now and then did he lift the right leg from the bed or move the fingers of the right hand; and upon directing my attention more particularly to these symptoms, I found that a partial paralysis of sensibility existed throughout the whole of the right half of the body, and that the muscles of the right arm, with the exception of those going to the fingers, were in a state of *rigid contraction;* so much so indeed that the limb could be bent at the elbow only by using considerable force. The sensibility of the arm was manifestly not entirely destroyed, for pinching the skin excited an effort to move the limb, as evinced by the contraction of the muscles of the corresponding shoulder. The right leg was relaxed, but when pinched or pricked was now and then feebly raised from the bed.

No fracture of the skull could be detected. Reaction having been in a measure established, as indicated by the character of the pulse, temperature of the surface, and other signs, I did not consider it necessary to repeat the stimulant which had been given the previous evening, but simply ordered cloths wrung out of cold water to be applied to the head, and the bed to be surrounded by curtains to shut out light and noise.

On the following morning I found the temperature of the skin, both of the extremities and trunk, quite hot but moist; pulse 120, soft and compressible; respiration somewhat hurried but easy; the bladder still emptying itself involuntarily without any discernible accumulation. I also learned that a large semi-liquid evacuation from the bowels had occurred during the night. There was, however, no reaction in the mental faculties, and the partial loss of sensibility in the right side, and the rigidity of the muscles of the right arm, continued.

The directions of the day before were repeated, with the addition of a large blister to the inner side of each thigh. The patient died, however, during the ensuing evening, fifty-three hours after the accident.

*Autopsy.*—The body was opened fourteen hours after death, the examination being limited to the head, as it was evident from the history of the case that this was the only part injured.

Upon lifting the brain, surrounded by the greater part of the dura mater, from its place, attention was first directed to the bones of the cranium, but no sign of fracture or other injury could be found, nor was there any effusion of blood between the dura mater and the bony surfaces. Removing the dura mater from the brain, the first abnormal appearance that I observed was a slight congestion of the veins of the pia mater, but no extravasation. Proceeding next to slice the brain carefully, commencing above, I could detect no unusual fullness of the vessels of the organ; but situated near the centre of the anterior lobe of the left hemisphere was a very small longitudinal rent containing a tolerably firm clot of blood about the size and very nearly the shape of a large pea. The clot was easily lifted from its bed, and, what was singular, the surrounding parts were scarcely stained by the effusion and not in the least congested. Continuing the dissection, I came upon a similar clot, but not larger than a No. 4 shot, in the posterior lobe of the right hemisphere, just beneath the gray layer.

*Remarks.*—The value of this case, both in a diagnostic and pathological point of view, will be readily appreciated. With the exception of the partial paralysis of the right side of the body, the symptoms were those of a pure but severe case of concussion. The exception in question was, however, sufficient to induce a strong suspicion of grave and probably fatal lesion to the central substance, and I so expressed myself from the time that I first saw the patient. As to the complete and protracted suspension of the intellectual powers, this is not unfrequently witnessed in cases of simple concussion, and, except when it continues for some time after entire reaction of the circulatory and secreting functions is not a very alarming symptom.

The post-mortem appearances account sufficiently, according to our present notions of the relations of the cerebral hemispheres to the two sides of the body, for the partial paralysis of the right arm and leg; but how is the rigidity of the right arm to be explained? This was a puzzling symptom to me, and is worthy the attention of physiologists.

*Concussion; Suspension of Intellectual Faculties for Four Days; Recovery.*

J. W., aged fourteen, was admitted into the hospital, October 8th, 1859, having been injured a few hours previously by being thrown upon the sod from the back of a mule. It could not be ascertained what portion of the body struck the ground first. The symptoms were those of an uncomplicated but severe case of concussion; heart's action feeble, and pulse correspondingly weak but slow, beating only 60 per minute; countenance pale, and features pinched; respiration natural; pupils unaltered and contracting promptly under the influence of light; vomiting and involuntary passage of urine. The intellectual faculties, as is usual in such cases, were not completely suspended; for when the body was forcibly shaken, a muttering complaint could be elicited, but no intelligent answer to the simplest question could be obtained, however loudly spoken in his ears.

Dr. Dismeger, who attended the case for two days before it fell into my hands, employed none of the ordinary means to bring about reaction, except the administration of a little brandy and water, which, being vomited, was followed by only partial success. At the time of my first visit, forty-eight hours after the accident, all the symptoms above mentioned were present except the paleness of the countenance, contraction of the features, and coldness of the extremities. As the functions of the body generally seemed to be in a tolerably good condition, and the symptoms not alarming, I did not consider it prudent to repeat the stimulants, but ordered the head to be placed low, perfect quiet, and the regular administration of milk and beef-tea every three or four hours.

On the following day the color of the face was decidedly better; extremities warm; pulse somewhat increased in force, but no improvement in the cerebral symptoms. Ordered blisters to the thighs, and continuance of regimen.

On the morning of the twelfth, four days from the receipt of the injury, the patient opened his eyes as though awaking from an ordinary sleep, looked around evidently in perfect ignorance of his situation, and being told where he was, tried to get out of bed in order to go home. Upon being questioned, he could give no account whatever of the accident, but remembered only having been upon the mule's back. By gentle confinement to bed, low regimen, and slight purgation added to the counter-irritation which had already been made by the blisters, inflammation of the brain was prevented, and in four days more the boy was allowed to be taken home, with directions to continue the treatment for a few days longer.

*Remarks.* The object in reporting the above case is to show the good effects of a more negative course of treatment than is usually pursued in such cases. It is true that the patient had taken brandy, but this had been ejected very soon after, and he subsequently took nothing whatever except milk and beef-tea. The great advantage which such a plan possesses is the security which it affords against inflammation of the brain. It is only where the prostration is excessive that a resort to stimulants is, in my opinion, at all justifiable, and I am convinced that if the majority of cases were let alone, we should have fewer deaths from subsequent inflammation.

*Compound Fracture of the Skull, with Depression of Bone and no Symptoms of Compression; Remarks upon the General Treatment of such Cases.*

During the month of February, 1860, three cases of compound fracture of the cranium, with depression of bone and no symptoms of depression, were admitted into my wards at the Charity Hospital. Of these, one was what Mr. Erichsen terms a "longitudinal punctured fracture," made with the edge of a hatchet; the second, an extensive compound comminuted fracture, with laceration of the brain, produced by a blow from a hammer; and the third, a compound fracture, with angular depression of bone, inflicted by a stone or brick. In the first case, the wound was closed by the house surgeon, by means of stitches, and the parts kept constantly cool and moist by the application of cloths wrung out of water. The patient recovered without a bad symptom. In the second case, inflammation of the brain, induced by the injury which the organ received at the time of the accident, supervened upon the third day, and death resulted at the end of a week. The third case is here reported in full for the purpose of illustrating the treatment which I usually pursue under such circumstances, in opposition to that of a large majority of American and British surgeons.

J. C., a large, well-built, hearty-looking Irishman, about thirty years of age, was struck upon the forehead by a stone or brick, he did not know which, on the night of the first of February. He fell to the ground in a state of insensibility, but soon recovering his consciousness, got up, and was led by his friends to the hospital, where he was put to bed, and cloths wrung out of cold water applied by the student of the ward. Examining the next morning, I found a contused, lacerated wound of the forehead, situated a little to the left of the median line, and an inch and a half above the orbital arch. The wound was sufficiently large to admit the end of the forefinger, upon introducing which, and also by exposing the parts to the light, I readily ascertained that there existed a fracture of the skull, with angular depression. Two irregular pieces of bone, each about as large as a ten-cent piece, had been driven in obliquely upon the brain, and were so firmly impacted as to be immovable by any ordinary force made with the finger or forceps. The depth of the depression at its lowest point below the level of the surrounding bone was nearly eight lines. There were also a few small splinters or chips of bone lying loose in the wound.

As already intimated, the man had no symptoms of compression, and, at the time of my visit, expressed himself as entirely comfortable, save a very slight headache. Under these circumstances, and as it was impossible to remove or even raise the depressed fragments without recourse to the trephine, I contented myself with simply picking out the loose bits, and directed the cold cloths to be continued, a rigid antiphlogistic diet, perfect quietude, and the administration of a dose of compound cathartic pills. Under this treatment, which, with the exception of the purgation, was continued from day to day, the wound gradually filled up from the bottom with healthy granulations, and in less than three weeks was entirely healed, the patient not having had a single untoward symptom. It is now four months since the accident, and although I have not seen the man since he left the hospital, as he has not sent for me nor applied again for admission, I have good reason to believe that he remains perfectly well.

*Remarks.*—My object in reporting this case, and in mentioning the other two in connection with it, is to show, in the first place, that compound depressed fractures of the skull without symptoms of compression are not very rare, Mr. Erichsen to the contrary notwithstanding; and, secondly, to furnish evidence in favor of modifying a generally received surgical dogma concerning their treatment. In regard to the latter question, if the reader will take the trouble to refer to the standard surgical works published in this country and in Great Britain, within the past half century, he will find that the trephine or the elevator is directed to be employed in all cases of compound fracture with depression, whether symptoms of compression exist or not. Take, for example, the recently published "System of Surgery," by Professor Gross, pronounced by Professor Simpson, of Edinburgh, to be the most complete treatise on surgery in the English language. The following is his language: "The proper treatment in compound fracture is to elevate the depressed bone and remove any loose or partially detached pieces, this plan being adopted whether there be any compression or not. The case being a compound one cannot be aggravated by operation, though it is not to be forgotten that this should be executed with the greatest care and gentleness." Mr. Erichsen is equally positive; and if we go back a few years to the great modern lights, such as Sir Astley Cooper and Mr. Abernethy, we find this same doctrine laid down as a law not to be questioned. Accustomed as the Anglo-Saxon race has been for so many centuries to obedience to law, departing from it only by slow degrees, as circumstances seem gradually to require, or breaking suddenly away from it only under the pressure of great emergencies, it is not strange that a doctrine backed up by such authority, and reaffirmed by nearly all subsequent teachers, should have all the force of an enactment of the Medes and Persians, which, we are told, altereth not. Now, with all due deference to the opinions and decrees of those who have established or perpetuated this rule, and without partaking in any manner whatever of the spirit or temper of the party who pleasantly style themselves "Young Physic," I am disposed to object to the wide range which has been given to this dogmatic precept. I have no objection to picking out pieces of bone which are completely detached from their connections with the dura mater and pericranium, or so nearly so that their little vitality must be inevitably lost in the subsequent reaction; nor would I hesitate to remove a jagged or splintered fragment that might be sticking through the dura mater into the brain; but I cannot understand the necessity of removing the bone simply because it is found resting upon the dura mater. To elevate it to its place, when this can be accomplished without further injury to the cranium, would be all right enough, but it is rarely feasible, the great majority of cases demanding for this purpose the use of the trephine.

If I understand the matter, the practice is grounded upon the fact that the depressed bone is necessarily a cause of irritation which will subsequently develop inflammation of the brain or its membranes, whether these structures be wounded or not. Upon this subject I have two suggestions to make.

In the first place, those who take this ground also advocate non-intervention in simple depressed fracture without symptoms of compression. Now if the depressed bone be a cause of inflammation of the encephalon in compound fracture, it is not less so in simple fracture. Indeed, this unfortunate result, according to these premises, would be more likely to occur in the latter than in the former, in consequence of the additional pressure exerted by the extravasated blood and the products of the subsequent inflammation of the subcutaneous structures. And this, I think, will be found to hold good in practice; that is to say, that inflammation of the brain and its membranes oftener occurs in simple fracture with depression than in compound, the same expectant treatment being employed in both.

Secondly, it is not true that pressure upon the brain induces inflammation of the organ, and in proof of this I need only refer to the fact that apoplectic effusions are seldom if ever followed by such an occurrence. The cause of inflammation of the brain after fracture I conceive to be the bruising, laceration, or jarring to which the organ has been subjected; and if such be the fact, the removal of the depressed bone will in no degree serve to prevent this much-dreaded result, except in those cases in which the fragments have penetrated the dura mater. I might even go still further and state, as has been suggested to me by my distinguished colleague, Professor Stone, that it is not altogether improbable that the removal or elevation of the depressed bone, independently of the injury done by the trephine, may even contribute to the establishment of inflammation by permitting the entrance of a greater amount of blood into the bruised part when reaction takes place than could occur if the pressure were allowed to continue.

In regard to the statement made by many writers, that the use of the trephine does not in any manner complicate a case of compound depressed fracture, I would simply ask whether that exposure of the dura mater which is what they so much fear in simple fracture as to advise against the use of the trephine, is not largely increased by the application of the instrument in compound fracture, to say nothing of the further injury to the bone? It is true that in times long gone by, and it is to be hoped never to return, when a perfect mania for trepanning prevailed among surgeons, large portions of the cranium are said to have been removed with evident advantage to the patient;* but this by no means proves the innocuousness of the operation.

Lastly, I would state, without fear of contradiction, that the number of persons who recover after compound fracture with depression, in whom the trephine is not employed, is far greater than of those who have been subjected to the operation. Common observation proves this beyond any doubt; but as additional evidence, I would refer the reader to the statistics of Mr. Lawrie and Mr. King, in Cormack's *Monthly Journal*, 1844, where it will be found that of 77 cases reported of compound fracture, there were 29 cures and 48 deaths; 26 of these 77 cases were not trephined, and of these 18 were cured and 8 died; 51 of the 77 cases were trephined, and of these 11 were cure and 40 died.†

The only question that remains to be considered in this connection is the liability to epilepsy after depressed fracture when the fragment of bone is allowed to remain in its unnatural position. The number of cases of epilepsy directly ascribable to this cause, compared with the total number of cases in which depressed bone is permitted to remain below the level of the surrounding surface, is acknowledged by all to be very small, and does not therefore usually enter into the discussion of the subject. But according to the views here enunciated concerning the propriety of using the trephine in the class of cases under consideration, the question becomes, in my opinion, of considerable importance. In the absence, however, of sufficient data, I am compelled to leave it for further investigation.

---

* It is mentioned, in John Bell's "Principles of Surgery," that Godifredus, chief surgeon to the States of Holland, was accustomed to boast that his friend Henry Chadborn, chirurgeon, during King William's wars on Philip, Count of Nassau, trepanned the skull of the count *twenty-seven* times, and substantiated the fact by the most indisputable authority, for he made the said Count of Nassau, after he was recovered, on the 12th day of August, 1664, write the following curious certificate: "I, the underwritten Philip, Count of Nassau, hereby declare and testify that Mr. Henry Chadborn did trepan me in the skull twenty-seven times, and after that did cure me well and soundly."

† Mott's Velpeau, vol. ii. p. 942.

*Subnitrate of Bismuth, in the Treatment of Burns and Scalds.*

To the thousand and one local remedies resorted to by medical men, as well as non-medical persons of both sexes, in the treatment of burns and scalds, I have the temerity to add another, if, indeed, I have not been anticipated by some one else. The substance referred to is the subnitrate of bismuth, which I employed during the past winter with remarkable success in the wards of the Charity Hospital. Heretofore, I was in the habit of using the white-lead and linseed oil, as recommended first by Professor Gross, in the March number of Dr. John Bell's *Medical Bulletin* for 1844, and, although I consider it far superior to any other application in common use, I am now convinced by ample experience that the bismuth is better. I was induced to give it a trial from a consideration of its well-known effect in calming irritation, and even actual inflammation occurring in mucous membranes, the condition of these structures under such circumstances bearing a very close analogy to that of the skin after a burn of the first or second degree.

When I first began its use, I combined it with linseed oil in such proportions as to form a consistent paint, but subsequently substituted glycerin for the oil, and I am now inclined to think that the combination can never be surpassed, since by it every local indication is fully met. To prepare it, it is only necessary to rub the bismuth in a mortar with a sufficient amount of glycerin to form a paste or thick paint, which should be applied to the affected surface by means of a camel's-hair pencil or a mop made of soft linen. Previously, however, to making the application, the parts should, if possible, be thoroughly dried, and for this purpose it is necessary to prick with a needle or other fine instrument any blisters that may exist, and carefully wipe the surface, by gently pressing upon it a piece of dry lint or charpie. A thick coating having then been put on, the parts should be protected from the pressure or friction of the bedclothes by covering it with a sheet of clean carded cotton or a layer of cotton batting, which may be confined, if necessary, by a thin bandage lightly applied. In burns of the first degree, one such application will often suffice, but in those of the second degree, it may be necessary to repeat it, in part at least, from day to day, in consequence of its disturbance and the wetting of the cotton by the subjacent discharges. The great object is to keep the denuded and tender surface entirely protected from atmospheric contact, which is especially important in the wards of hospitals where the air is always more or less loaded with noxious gases.

In slight burns of the first degree in which there is only an erythematous inflammation without any vesication, the glycerin may be conveniently dispensed with and the dry bismuth dusted thick over the surface, in which case the watery exudations from the skin will form with it a pasty coating sufficiently protective. But still the glycerin, possessing of itself properties powerfully soothing in their influence, should always be combined with it, unless there be some reasonable objection to its use.

The carded cotton or cotton battery I look upon as a most valuable adjuvant, and is superior to anything with which I am acquainted for warding off pressure.*

* Since writing the above, Dr. W. C. Nichols, the highly accomplished House Surgeon of the Charity Hospital, has informed me that he has given the bismuth and glycerin a fair trial in a number of cases of burns under his charge, and freely accords to it all the praise which my experience has elicited in its behalf.

